# The Brazilian Portuguese version of the Pregnancy Mobility Index: Cross-cultural adaptation and psychometric evaluation – a validation study

**DOI:** 10.1590/1516-3180.2022.0279.R1.19122022

**Published:** 2023-05-08

**Authors:** Maria Izabel Feltrin, Rubneide Barreto Silva Gallo, Elisa Gabardo Lima, Nayara Helena Gomes Bertoncini, Jordana Barbosa da Silva, Natália Boneti Moreira, Raciele Ivandra Guarda Korelo

**Affiliations:** I Physiotherapist, Department of Prevention and Rehabilitation in Physical Therapy, Universidade Federal do Paraná (UFPR), Curitiba (PR), Brazil.; II MSc, PhD. Professor, Department of Prevention and Rehabilitation in Physical Therapy, Universidade Federal do Paraná (UFPR), Curitiba (PR), Brazil.; III Physiotherapist, Department of Prevention and Rehabilitation in Physical Therapy, Universidade Federal do Paraná (UFPR), Curitiba (PR), Brazil.; IV Physiotherapist, Department of Prevention and Rehabilitation in Physical Therapy, Universidade Federal do Paraná (UFPR), Curitiba (PR), Brazil.; V MSc. Physiotherapist and Doctoral Student, Women’s Health Research Laboratory, Universidade Federal de São Carlos (UFSCar), São Carlos (SP), Brazil.; VI PhD. Professor, Department of Prevention and Rehabilitation in Physical Therapy, Universidade Federal do Paraná (UFPR), Curitiba (PR), Brazil.; VII MSc, PhD. Physiotherapist and Adjunct Professor, Department of Prevention and Rehabilitation in Physical Therapy, Universidade Federal do Paraná (UFPR), Curitiba (PR), Brazil.

**Keywords:** Pregnant women, Mobility limitation, Women’s health services, Lumbosacral region, Women’s health, Pregnancy validation studies, Lumbar region, Health, women’s

## Abstract

**BACKGROUND:**

The Pregnancy Mobility Index (PMI) was developed to assess mobility in pregnant women in the Netherlands. At present, no similar questionnaire is available in Brazil.

**OBJECTIVE:**

The present study aimed to translate, cross-culturally adapt, and evaluate the psychometric properties of a Brazilian PMI.

**DESIGN AND SETTING:**

The present study was a validation study conducted at the Universidade Federal do Paraná and a public maternity ward in Curitiba, Brazil.

**METHODS:**

Text translation and cross-cultural adaptation followed international guidelines. Construct validity, internal consistency, and inter- and intra-rater reliability tests included 97 women. The Pelvic Girdle Questionnaire, Multidimensional Pain Evaluation Scale, Schober’s test, and lumbar spine range of motion assessment were administered on the first day. Intra-rater reliability (n = 19) was measured after 15 days. Exploratory factor analysis was performed, and the correlation matrix was analyzed using Pearson’s coefficient.

**RESULTS:**

Pregnant women (88%) understood the cultural adaptation process. The internal consistency was high (Cronbach’s alpha > 0.90), construct validity was moderate, with significant correlation between lumbar spine range of motion (r = 0.283–0.369) and Schober’s test (r = -0.314), and high correlation between the Multidimensional Pain Evaluation Scale (r = -0.650 and -0.499) and Pelvic Girdle Questionnaire (r = -0.737). Intra- and inter-rater reliabilities were excellent (intraclass correlation coefficient = 0.932 and 0.990, respectively).

**CONCLUSION:**

The Brazilian version of the PMI was successfully translated with excellent reliability and moderate-to-high construct validity. It is an important tool for assessing mobility in pregnant women.

**CLINICAL TRIAL:**

RBR-789tps (Validation study), https://ensaiosclinicos.gov.br/rg/RBR-789tps.

## INTRODUCTION

The anatomical and physiological changes that occur during pregnancy frequently increase musculoskeletal disorders. Symptoms frequently related to pregnancy are largely due to ligamentous laxity and joint hypermobility, which are associated with hormonal changes and weight gain. This, in turn, increases mechanical stress. Additionally, pregnant women have a displaced center of gravity, which is associated with hyperlordosis that contributes to the mechanical strain on the sacroiliac and back joints.^1,2^ Low back and pelvic girdle pain are the most frequent complaints during pregnancy, and both negatively affect mobility and functionality, contributing to physical disability that can affect work performance and day-to-day activities.^3,4^

Although there are several questionnaires that evaluate disability and loss of mobility caused by back pain in the general population, none are specific to pregnant individuals.^3,4^ Pregnancy-related back pain differs from that in the general population, and has distinctive mobility patterns and expectations.^4^ A questionnaire is a tool that transforms subjective information into objective and measurable data. In this way, it is possible to demonstrate the patient’s evolution to them in a clearer and more understandable way. The advantage of using questionnaires is that they are self-reported by the patient or the healthcare worker, in research or the clinical setting. In clinical practice, questionnaires can become a facilitator for medical records, assisting the healthcare professional in understanding the patient’s needs and, later, in the execution of the treatment plan.^5^

In Brazil, there is only one questionnaire available with which to assess the interference of lumbopelvic pain in sexual activity, sleep quality, and day-to-day activities during pregnancy.^3^ As of yet, there is no questionnaire that assesses the mobility of pregnant women which has been validated for the Brazilian population;^3,4^ however, the Pregnancy Mobility Index (PMI) could fill this gap, as it was developed to specifically assess this variable.^4^ The PMI, composed of 24 questions which aim to assess the effects of low back and pelvic pain during and after pregnancy on day-to-day activities, is considered a reliable and valid instrument when applied to the Dutch population. The PMI can also assess the effects of pain interventions and help understand normal levels of mobility during pregnancy.^4^

## OBJECTIVE

The aim of the present study was to provide a cross-cultural adaptation and psychometric evaluation of a Brazilian Portuguese version of the PMI.

## METHODS

The present validation study, performed using the Consensus-Based Standards for the Selection of Health Measurement Instruments (COSMIN) guidelines,^6^ was approved by the ethics committee at the Universidade Federal do Paraná (UFPR) (Number:2.399.033; CAAE 78877417.8.0000.0096; approved in 2017) and registered in the Brazilian Registry of Clinical Trials as a validation study (RBR-789tps - https://ensaiosclinicos.gov.br/rg/RBR-789tps). The present study was conducted in Curitiba, Brazil, at the Victor Ferreira do Amaral Hospital and Maternity Ward, and at the Prevention and Rehabilitation in Physical Therapy Department of the UFPR. All individuals provided written informed consent prior to participating in the study.

To begin, we requested authorization for the translation and validation of a Brazilian Portuguese version of the PMI. The Dutch and English versions of the PMI were utilized for the preparation of the Brazilian version.^4,7^ The process of translation, back translation, and cross-cultural adaptation rigorously followed the Guidelines for the Process of Cross-Cultural Adaptation of Self-Report Measures.^8^

Four bilingual translators, all native speakers of Brazilian Portuguese, translated the instrument; two were healthcare professionals (T1D and T1E), while the other two had no healthcare experience (T2D and T2E). The translations were discussed by the translators and the authors of the present study, and the first version of the translated PMI was created (T-12D and T-12E). This version was translated back to English and Dutch by four native speakers, none of whom were healthcare professionals or had prior knowledge of the original version of the PMI (BT1D, BT2D, BT1E, and BT2E). To reach a consensus, a committee of experts reviewed a document containing the translations, back-translations, original versions, and a report prepared by the research team, including each item of the instrument, alternative answers, and instructions. The committee’s decisions were aimed at ensuring semantic, idiomatic, experimental, and conceptual equivalence between the versions. The pre-final version was administered to 30 eligible participants to evaluate for any difficulties in item comprehension.

In order to verify the comprehension of the instrument by the participants, we followed the steps described in a previous study.^9^ Pregnant women were questioned about the comprehension of each item, and answers were based on a scale that ranged from 0–5. Additionally, participants were instructed to answer three open-ended questions. The research team calculated the percentage of understanding, and the committee verified that all recommended steps had been followed. The final version of the Brazilian Portuguese PMI was created.

The Brazilian Portuguese PMI has 22 questions using the following 3 subscales (Appendix): daily mobility in the home; household activities; and mobility outdoors. Each question regarding the limitations of the lumbopelvic region in performing the activities was scored on 4-point Likert scale (0 = no difficulty; 1 = little difficulty; 2 = very difficult; and 3 = impossible to perform without help). Additionally, it was also possible to choose “not applicable” as an answer (this item, when checked, was not used to calculate the final score).

After the translation and cross-cultural adaptation, validity and reliability were assessed at a public maternity hospital. Primiparous and multiparous pregnant women at a gestational age > 20 weeks, without cognitive deficits, and who were native Brazilian Portuguese readers were included. Women were excluded if they had a high-risk pregnancy (twins, triplets, or more pregnancies and/or with diabetes, hypertension, measles, rubella, and/or a urinary tract infection), had psychiatric and/or neurological disorders, and/or were unable to perform the tests.

Sociodemographic data were collected during the first interview, including age, gestational age, ethnic characteristics (Caucasian, African, Asian, multiple ethnicities), marital status (single, married, divorced), level of education (primary incomplete/complete, secondary complete, college complete), occupational status (employed, homemaker), lifestyle (smoking habits, alcohol consumption, physical activity), and presence of low back pain before and during pregnancy. After the researchers collected the data, the Brazilian Portuguese PMI was administered three times (at the beginning of the interview, after 30 minutes, and after 15 days), per the guidelines provided by the COSMIN initiative.^10^

The participants were asked to answer the Brazilian Portuguese PMI multiple times – first at the beginning of the interview (Examiner 1) and again after 30 minutes (Examiner 2). In the same interview, lumbopelvic incapacity was evaluated using the Pelvic Girdle Questionnaire (PGQ),^11^ pain intensity was evaluated using the Multidimensional Pain Evaluation Scale,^12^ and lumbar spine range of motion was evaluated using the modified Schober’s test^13^ and fleximetry.^14^

In the second interview, which was arranged on average 15 days after the first interview, the PMI was completed again, by 19 pregnant women who had already answered the PMI at the first assessment, via a posting by Examiner 1 on a mobile instant messaging service. The participants did not receive any interventions or treatment for low back or pelvic pain during the study period.

In the present study, we made changes in the following: 1) the way the questions were described (statement); 2) we added an answer option (“not applicable”) to the questions; and 3) we added a scoring formula to the questionnaire, which guarantees a consistent calculation of the final score. Semantic and cultural adaptations provided the greatest comprehension of the questionnaire.

The final score was calculated by adding the score obtained for each question, multiplying by 100, and dividing by the number of questions scored multiplied by 3. The final scores ranged from 0–100, where 0 equaled ‘normal performance’ and 100 indicated ‘maximum disability’, and consisted of the mobility index of the pregnant woman, as formulated below.


$$Mobilityindex=\frac{\left[100-(\;sumofthescoresobtained\;)\times 100\right]}{\;numberofquestionsscored\;\times 3}$$

### Statistical analysis

Descriptive statistics (mean and standard deviation for continuous data, and frequency and percentages for categorical data) were analyzed to characterize the participants. All analyses were performed using a 95% confidence interval (CI). The intraclass correlation coefficient (ICC) and Bland-Altman method^15,16^ were utilized to evaluate the inter- and intra-rater reliability and concordance of the PMI, respectively. ICCs were interpreted as follows: poor (< 0.4); fair (0.4–< 0.6); good (0.6–< 0.75); and excellent (≥ 0.75).^15^ The factor analysis followed the main component analysis with Varimax rotation. To analyze the internal consistency, a standardized Cronbach’s alpha coefficient was utilized. The Pearson coefficient was utilized to evaluate the construct validity of the PMI between other instruments and tests (PGQ, Multidimensional Pain Evaluation Scale, Schober’s test, and lumbar spine range of motion), and the coefficients were interpreted as follows, based on the magnitude scale proposed by Hopkins:^17^ trivial (< 0.1); small (0.1–0.29); moderate (0.30–0.49); large (0.50–0.69); very large (0.70–0.90); and nearly perfect (> 0.90). Statistical analyses were performed using the Statistical Package for the Social Sciences (SPSS) software, version 22 (IBM, SPSS Inc., Chicago, IL, United States), and the significance level was set at P < 0.05.

The sample size was determined according to guidance from Terwee et al.,^18^ which suggested a ratio of ≥ 4–10 participants for each instrument containing 24 questions. A total of 106 pregnant women participated in the present study, although 9 were excluded because they were either high-risk (n = 6) or failed to complete the proposed tests (n = 3). The final validation sample included a total of 97 pregnant women.

## RESULTS

Some discrepancies between the original version and those analyzed by the committee were observed during the translation and back-translation processes. These discrepancies were resolved using strategies such as word addition, omission, or substitutions to search for semantic, conceptual, idiomatic, and experimental equivalence. Using these strategies, it was possible to generate equivalent expressions in Brazilian Portuguese. All modifications were performed prior to pretesting. The cross-cultural adaptation involved 30 women, and high comprehension (88%) of all items of the pre-final version was observed, which indicated no further need for revisions.

The validation phase included a total of 97 pregnant women. The mean age was 26.8 ± 6.2 years, and the intensity of the low back pain was considered light before pregnancy and advanced-to-moderate during pregnancy. The results are shown in Table 1.

**Table 1:** Characteristics (frequency and percentage) of the study sample (n = 97)

Continuous characteristics		Mean	SD
**Age (years)**		26.8	6.2
**Gestational age (weeks)**		31.7	6.2
**Low back pain intensity (points)**			
	Before pregnancy	1.6	2.7
	During pregnancy	5.7	2.4
**Categorical characteristics**		n	%
**Ethnic**			
	Caucasian	59	60.8
	Mixed ethnicity	21	21.6
	African	13	13.4
	Asian	4	4.1
**Marital status**			
	Married	52	53.6
	Single	39	40.2
	Divorced	6	6.2
**Educational level**			
	Primary incomplete	7	7.2
	Primary complete	12	12.4
	Secondary complete	56	57.8
	College complete	22	22.7
**Occupational status**			
	Housewife	25	25.8
	Administrative assistant	23	23.7
	Businesswoman	13	13.4
	Student	11	11.3
	Healthcare professional	9	9.3
	Maid	8	8.2
	Saleswoman	6	6.2
	Teacher	2	2.1
**Lifestyle**			
	Smoking habits	6	6.2
	Alcohol consumption	0	0
	Physical activity practice	12	12.4
**Presence of low back pain**			
	Before pregnancy	30	30.9
	During pregnancy	92	94.8
	Only during pregnancy	62	64.8

SD = standard deviation; n = number.

**Figure 1A-B:**
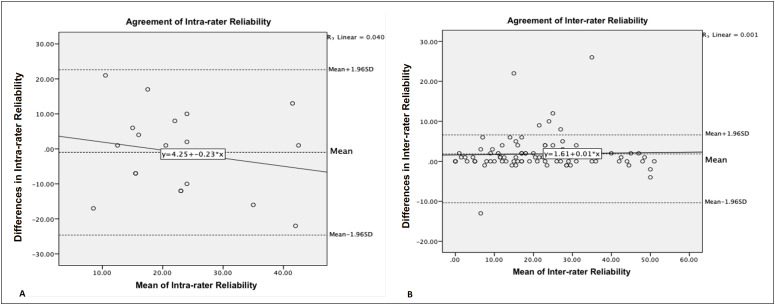
Bland-Altman Intra- and Inter-rater Reliability graphic.

The reliability and concordance results of the intra- and inter- rater reliability tests, which are shown in Table 2, indicated high intra- and inter-rater reliability (ICC = 0.93 and 0.99, respectively). The paired-samples Student’s t-test did not show significant differences in the average test-retest scores for intra-examiner reliability (P = 0.722), although it was different (P = 0.000) from inter-examiner reliability. Bland-Altman plots (Figures 1A–B), however, revealed a mean error in the difference between the intra- (1.00, standard deviation, SD = 12.06, 95% CI = -24.63–22.63) and inter-examiner reliability (1.85, SD = 4.32, 95% CI = -10.35–6.60) close to zero. The P-value of the regression analysis showed that the slope of the curve did not deviate from zero (intra-examiner reliability, P = 0.412; inter-examiner reliability, P = 0.741). Therefore, these results represent 95% agreement between the test and retest scores.

**Table 2:** Results of reliability and concordance of intra- and inter-rater test (n = 96)

	ICC		Bland-Altman			95% LoA	
	ICC	95% CI	$\bar{d}$	95% CI of $\bar{d}$	SD of $\bar{d}$		
Intra-examiner	0.93	0.78; 0.97	-1.00	-6.81; 4.81	12.06	-24.63	22.63
Inter-examiner	0.99	0.97; 0.99	1.85*	0.98; 2.72	4.32	-10.35	6.60

ICC, intraclass correlation coefficient; CI, confidence interval; LoA, limits of agreement; $\bar{d}$ = bias, the difference between the two measures; SD, standard deviation; SD of $\bar{d}$ , standard deviation mean difference;

* P < 0.05, paired sample *t*-test.

The suitability of the scale was evaluated using factor analysis. The Kaiser-Meyer-Olkin value vas was 0.857, and the Bartlett sphericity test (χ 2 = 1232.79; d.g. [degrees of freedom]: 210;P < 0.000) indicated that the data was adequate for conducting the factorial analysis.

The factor analysis included 22 questions from the original PMI; however, questions 17 (“traveling by train”) and 19 (“traveling by bicycle”) were removed because they did not apply to the study population. The Brazilian Portuguese version of the PMI extracted three components, as did the original PMI. After the removal of an item that presented an unsatisfactory factorial load (Item 4), the three components showed a delimitated factorial distribution, each with at least four questions, presenting self-values > 1 (Table 3). A cut-off value of 0.40 was applied for the factor loadings, both in relation to the proximity of the items in the analysis and adherence to the theory. These factors corresponded to the subscales for daily mobility in the house (eigenvalue = 5.503), household activities (eigenvalue = 4.757), and mobility outdoors (eigenvalue = 4.691), which explained 71.20% of the variance. The model presents limitations related to cross-loading, which was < 0.20 for questions 7, 13, 14, and 18. The maintenance of the model’s structure was chosen because of the higher loads in the question origin factor and the expected correlation between the scale factors. These results indicate the reliability of the PMI, meaning that the Brazilian Portuguese version of the questionnaire was able to accurately evaluate mobility during pregnancy. Furthermore, each subscale had high internal consistency (0.933, 0.911, and 0.907 for the first, second, and third components, respectively), with Cronbach’s alpha above the recommended value.^18^

**Table 3:** Factor and principal component analysis with Varimax rotation

		Component 1^a^	Component 2^b^	Component 3^c^
**Question 1**			0.725	
**Question 2**			0.882	
**Question 3**			0.882	
**Question 5**			0.735	
**Question 6**			0.812	
**Question 7**			0.496	0.665
**Question 8**		0.760	0.423	
**Question 9**		0.708		
**Question 10**		0.704		
**Question 11**		0.814		
**Question 12**		0.849		
**Question 13**		0.605		0.471
**Question 14**		0.687		0.494
**Question 15**		0.745		0.439
**Question 16**				0.579
**Question 18**			0.476	0.549
**Question 20**				0.615
**Question 21**				0.795
**Question 22**				0.825
	Eigenvalues	5.503	4.757	4.691
	Variance explained (%)	26.21	22.65	22.34
	Cronbach’s alpha coefficient	0.933	0.911	0.907

a Daily mobility in the house;

b Household activities;

c Mobility outdoors.

The PMI construct validity was established through Pearson’s coefficient with gestational age and tests (PGQ, Multidimensional Pain Evaluation Scale, Schober’s test, and lumbar spine range of motion) which ranged between -0.737 and 0.369. The results are presented in Table 4.

**Table 4:** Pregnancy Mobility Index construct validity between other variables, instruments, and tests

Variables and tests		Pearson’s coefficient
**Gestational age**		-0.173^a^
**Low back pain intensity**		
	Before pregnancy	0.027
	During pregnancy	-0.591^b^
**Pelvic Girdle Questionnaire**		-0.737^b^
**Schober’s test**		-0.314^b^
**Lumbar spine range of motion**		
	Flexion	0.325^b^
	Extension	0.283^b^
	Right lateral flexion	0.347^b^
	Left lateral flexion	0.369^b^
	Right rotation	0.161
	Left rotation	0.162
**Multidimensional Pain Evaluation Scale**		
	Acute pain intensity	-0.650^b^
	Chronic pain intensity	-0.499^b^

a P < 0.05;

b P < 0.01.

## DISCUSSION

The main findings of the present study are related to the translation and cross-cultural adaptation of the Brazilian Portuguese version of the PMI. The results of the present study indicate that the Brazilian Portuguese PMI is reliable, consistent, and can discriminate between regular and irregular mobility.

The translation, validity, and reliability process should be rigorously followed, as the assessment tools must be precise, objective, and of high quality.^8,18^ The present study carefully followed guidelines specifying how to perform a psychometric evaluation of a questionnaire,^19^ based on suggestions regarding the use of guidelines for the cross-cultural adaptation of patient-reported outcome measurements.^8^

Other studies reinforce the importance of ensuring the equivalence of the items of the translated questionnaire with descriptors of the original and translated instrument.^8,18^ This equivalence, however, is not only due to the direct and literal translation of the questionnaire, but also the necessary adjustment of each question of the instrument, to ensure that each measurement objective is preserved in a new culture.^8,18^

During the process of converting the PMI questionnaire to a Brazilian Portuguese version, it was necessary to remove three items from the original questionnaire. The first question (Question 4) was excluded using principal component analysis. The other two items (Questions 17 and 19) were excluded because they did not provide sufficient relevant answers for the analysis. In Brazilian culture, is it not common to travel by bicycle or train, unlike in other countries such as the Netherlands. Therefore, the Brazilian Portuguese version of the PMI included only 21 questions, compared to 24 in the original version.

The results of the present study showed a high internal consistency (Cronbach’s alpha > 0.90) for the Brazilian Portuguese PMI, indicating that the items of the instrument correlate with both the other items and the final score. This metric, therefore, shows an aspect related to reliability.^18,19^ Reliability was considered excellent for the intra- and inter-rater assessments (> 0.75), indicating that a set of PMI questions can evaluate pregnancy mobility, with similar results when the same respondents are assessed on different occasions without undergoing any change in health.^10^

Interestingly, the data showed that there was a reduction in the mean score (21.30) as measured by Examiner 2 after 30 min, compared to Examiner 1 (23.17, mean difference 1.85, P = 0.000). The reduction in mobility could be justified by the fact that pregnant women underwent clinical tests to evaluate mobility and range of motion (Schober’s test and fleximetry), demonstrating that they would be able to perform the clinical tests, contrary to their previous judgment as expressed in the PMI questions. In contrast, no systematic measurement error (P = 0.722) was found during the intra-rater reliability assessment performed 15 d after the first evaluation. One possible justification for this result may be related to the fact that pregnant women may have returned for their follow-up while retaining their initial perception of mobility, considering that the 3^rd^ administration of the questionnaire did not precede mobility and range of motion tests. Despite these findings, the Bland-Altman agreement analysis revealed that there were no systematic and/or random errors in the PMI scores attributed to true changes in mobility, as seen in Figures 1A-B, ensuring the reproducibility and concordance of the PMI.

The analysis of the construct validity between the Brazilian Portuguese PMI and other assessments (PGQ,^3^ Multidimensional Pain Evaluation Scale,^12^ Schober’s test,^13^ and lumbar spine range of motion^14^) indicated high and moderate correlations, proving the effectiveness of the Brazilian Portuguese PMI in evaluating mobility. During pregnancy, women undergo several physiological changes that impair mobility, which are usually enhanced by the presence of pain,^3,20–22^ justifying the greater correlation of the PMI with instruments that measure pain and reinforce the instrument’s quality of construct validity.

Pain is one of the predictors of mobility limitations during pregnancy, especially low back and/or lumbopelvic pain.^21^ Previous studies^21,23^ have identified that women at advanced gestational ages have a higher rate of low back pain (visual analog scale = 7, moderate-to-intense) and, consequently, greater mobility limitation in day-to-day activities. These limitations can subsequently affect the emotional state of pregnant woman.^23^ Corroborating these findings, the pregnant women assessed in the present study had more intense lower back pain in the third trimester of pregnancy than the first or second. The greatest degree of limitation, however, occurs in the second trimester, as previously demonstrated by Bakker et al.^23^

Data analysis revealed a high prevalence of lower back pain during pregnancy, and it is widely known that pain and discomfort have a significant impact on the daily, domestic, and work activities of pregnant women.^21,22^ Moreover, health education during pregnancy is an important tool to avoid inadequate movements in daily activities, and can be useful to prevent complaints about increased levels of pain.^24^ The PMI proved to be a tool that could help health professionals identify inadequate movements, considering that the instrument individually points out the difficulty of movement execution. In a prospective cohort study,^23^ 223 pregnant women in the Netherlands were followed from the 12^th^ to 36^th^ week of gestation, and the results supported the use of PMI to evaluate physical factors that can help in the prevention of significant pain.

One limitation of the present study is the high educational level of the sample population, which does not correspond to the profile of pregnant women from public hospitals in Brazil,^25^ highlighting the importance of replicating this instrument for various regions of the country. Patient profiles may be related to the location of the hospitals, which tend to be in the neighborhoods in the city with the highest Human Development Index ([HDI] 0.956) in the city. All participants in the present study, however, completed the questionnaire by themselves without receiving help from the interviewer, which may confirm the clear and simple description of the questionnaire, ensuring that all women, regardless of their educational level, were able to use this questionnaire. Another limitation of the present study is the absence of a relevant evaluation of women during the postpartum period, which is included in the original version of the PMI.^3^ Nonetheless, the main objective of the present study was to include pregnant women in the sample population. Therefore, we recommend that future studies should investigate the psychometric properties of the Brazilian Portuguese version of the PMI in the postpartum population. One strength of the present study is that the PMI presented high internal consistency and reliability, corroborating the original version. These results showed that the Brazilian Portuguese version of the PMI is adequate for detecting changes in mobility related to low back and pelvic pain in pregnant women. Additionally, the questionnaire may be utilized during research and clinical practice to assist and promote health education among pregnant Brazilian women.

## CONCLUSION

The Brazilian Portuguese PMI has been shown to be a reliable and valid questionnaire for use during pregnancy to evaluate and assist pregnant women in Brazil. The translation, cross-cultural adaptation, and psychometric evaluation of the Brazilian Portuguese PMI were successfully completed, and will contribute to health professionals’ clinical decisions, as the PMI is an important tool to assess the mobility of pregnant women in Brazil.

## Appendix Pregnancy Mobility Index – Brazilian Portuguese version.

Por favor, assinale com um “X” a opção mais adequada para cada item/atividades do quadro abaixo, relacionada a alguma queixa ou limitação em sua pelve e/ou coluna lombar.

Todos os itens/atividades têm pontuação de 0-3, conforme:

0 - Nenhuma dificuldade ou esforço para a realização da atividade;

1 - Um pouco de dificuldade ou esforço para a realização da atividade;

2 - Muita dificuldade ou esforço para a realização da atividade;

3 - É impossível realizar a atividade sem ajuda de outros

Caso nenhuma das opções satisfaça a sua resposta e/ou você não realiza a atividade questionada, você deve assinalar como “não se aplica”.

Nenhum dos itens/atividades pode ficar sem resposta.

**Table:**

Você vivencia alguma queixa ou limitação em sua pelve e/ou coluna lombar realizando as seguintes atividades?		Nenhuma (0)	Um pouco (1)	Bastante (2)	Precisa de ajuda (3)	Não se aplica
**Na movimentação diária em casa**						
	1. Levantando-se de uma cadeira					
	2. Levantando-se de um sofá					
	3. Levantando-se da cama					
	4. Colocando os sapatos					
	5. Virando-se na cama					
	6. Levantando-se do chão					
***Realizando atividades domésticas?***						
	7. Limpando com o aspirador de pó/vassoura/esfregão					
	8. Lavando roupas					
	9. Colocando roupas para secar					
	10. Na posição ajoelhada					
	11. Na posição agachada					
	12. Na posição em pé					
	13. Levantando 5 kg					
	14. Levantando 10 kg					
	15. Subindo ou descendo escadas					
***Atividades fora de casa:***						
	16. Viajando de carro					
	17. Viajando de ônibus					
	18. Caminhando 50 metros					
	19. Caminhando 200 metros					
	20. Caminhando 500 metros					
	21.Caminhando em uma superfície irregular					

**Cálculo:** as questões marcadas em ‘não se aplica’, não serão consideradas para o cálculo de incapacidade, devendo ser anuladas. A pontuação final varia de 0 a 100 pontos, em que 0 indica “capacidade normal” e 100 indica “máxima incapacidade”. Índice de mobilidade = [100 - ( pontuação obtida ) x 100] número de questões pontuadas x 3

$$Índicedemobilidade=\frac{\left[1-(pontuaçãoobtida)x1\right]}{númerodequestõespontuadasx3}$$
